# Association between body roundness index trajectories and the incidence of diabetes mellitus: a perspective from the China health and retirement longitudinal study

**DOI:** 10.1186/s12944-025-02840-y

**Published:** 2025-12-30

**Authors:** Fucun Ma, Ruixue Zhang, Wenyao Geng, Zheng Gao, Chenhui Li, Jie Liu, Jie Zhang, Xuekai Liu, Meijing Feng, Mingjian Bai, Guowei Liang

**Affiliations:** 1 Department of Clinical Laboratory, Aerospace Center Hospital, No. 15 Yuquan Road, Haidian District, Beijing, 100049 China; 2 Department of Health Management, Aerospace Center Hospital, Beijing, 100049 China

**Keywords:** Body roundness index, Diabetes mellitus, Trajectory analysis, Risk prediction, China health and retirement longitudinal study

## Abstract

**Objective:**

To investigate the associations between longitudinal body roundness index (BRI) trajectories and the risk of incident diabetes mellitus (DM) using data from the China Health and Retirement Longitudinal Study (CHARLS).

**Methods:**

Group-based trajectory modeling (GBTM) identified distinct BRI trajectories (Waves 1–3, 2011–2016). Their associations with DM incidence (Wave 4, 2017–2018) were assessed using multivariate *Cox* models. The predictive performance of a single baseline BRI was compared with body mass index (BMI) and waist circumference (WC) via receiver operating characteristic (*ROC*) analysis. Net reclassification improvement (NRI) and integrated discrimination improvement (IDI) evaluated the incremental value of adding BRI trajectories to a conventional risk model. Subgroup and sensitivity analyses, including a landmark approach, assessed robustness.

**Results:**

Among 4,150 participants, 103 developed DM. Three stable BRI trajectories were identified: low-stable (49.0%), moderate-stable (41.3%), and high-stable (9.7%). Compared with the low-stable group, the high-stable group had a significantly increased DM risk with a fully-adjusted hazard ratio (*HR*) of 2.63 (95% confidence interval [*CI*]: 1.41–4.91). A single baseline BRI showed comparable discrimination to BMI and WC (AUC ≈ 0.63). Longitudinal trajectories of BRI, BMI, and WC all identified high-stable subgroups with elevated risk (*HR*s: BRI = 2.63, BMI = 2.16, WC = 2.31), with overlapping confidence intervals. However, adding BRI trajectories to a conventional model significantly improved risk reclassification (NRI = 10.76%, 95% *CI*: 2.40–19.47) and discrimination (IDI = 0.27%, 95% *CI*: 0.03–0.52). Results were consistent across subgroups and sensitivity analyses.

**Conclusions:**

Sustained high BRI exposure, captured by longitudinal trajectory modeling, is independently associated with increased DM risk. While BRI trajectories were not statistically superior to BMI or WC trajectories, the longitudinal framework itself adds value over single-time-point assessments by more robustly identifying individuals with persistent high adiposity-related risk, highlighting the utility of monitoring long-term body shape stability for early risk stratification.

**Supplementary Information:**

The online version contains supplementary material available at 10.1186/s12944-025-02840-y.

## Introduction

 Diabetes mellitus (DM) constitutes a major global public health challenge, with its prevalence having quadrupled since 1990 and imposing a significant disease burden worldwide [1, 2]. This surge is intrinsically linked to the parallel epidemic of obesity [3, 4]. In China, the burden of DM is particularly severe, characterized by a continuously rising incidence and prevalence that imposes substantial strain on the healthcare system [5].

The assessment of obesity, a key modifiable risk factor for DM, relies on various anthropometric indices. Body mass index (BMI) and waist circumference (WC) are widely used but have recognized limitations; BMI does not distinguish fat distribution, and WC fails to account for height. The body roundness index (BRI), derived from a geometrical model based on height and WC, was developed to more accurately reflect visceral adiposity and body shape [6]. Emerging cross-sectional evidence suggests that BRI may outperform traditional indices like BMI, a body shape index (ABSI), and WC in identifying individuals at risk for DM and prediabetes [7, 8].

However, existing research still faces a key methodological limitation. Most studies, including those on BRI, are cross-sectional in design and have not fully explored the associations between changes in variables over time and disease onset [7, 9, 10]. This approach captures risk at a single time point but cannot elucidate how the long-term dynamic changes in body shape influence DM risk. The trajectory of an obesity indicator may provide more profound insights into disease etiology than a static measurement. Group-based trajectory modeling (GBTM) is a powerful statistical tool designed to identify latent subgroups of individuals following similar longitudinal patterns, thereby capturing heterogeneity and enhancing risk stratification [11–13]. Therefore, leveraging the longitudinal data from the China Health and Retirement Longitudinal Study (CHARLS), this study aims to transcend the limitations of cross-sectional analysis. We applied GBTM to characterize the long-term trajectories of BRI among middle-aged and older Chinese adults. Our primary objective was to investigate the association between distinct BRI trajectories and the subsequent risk of incident DM, and to evaluate whether this longitudinal approach provides incremental value over conventional single-time-point assessments using BRI, BMI, or WC.

## Materials and methods

### Study population

The participants of this study were drawn from the CHARLS, which focuses on middle-aged and older adults in China. CHARLS is a nationally representative and ongoing cohort study designed to assess the social, economic, and health conditions of the elderly population. The sampling process for CHARLS involves a multistage stratified cluster sampling method. During the baseline survey conducted between 2011 and 2012 (Wave 1), a total of 17,708 individuals aged 45 years and above from 10,257 households across 150 districts within 28 provinces were initially included. The participants in the CHARLS subsequently underwent biennial face-to-face computer-assisted personal interviews to ensure that the dataset remained updated. After the initial survey, four additional rounds of follow-up investigations were carried out among the surviving participants in the following periods: 2013–2014 (Wave 2), 2015–2016 (Wave 3), and 2017–2018 (Wave 4) [14]. All physicians involved in the study received training from the CHARLS staff at Peking University. Moreover, the study obtained ethical approval from the Biomedical Ethics Review Committee of Peking University for two reasons: the main household survey (including anthropometrics), with approval number IRB00001052–11,015, and biomarker collection, with approval number IRB00001052–11,014. All participants provided written informed consent. For comprehensive information regarding the CHARLS data, it can be accessed on its official website: http://charls.pku.edu.cn/en.

**Fig. 1:**
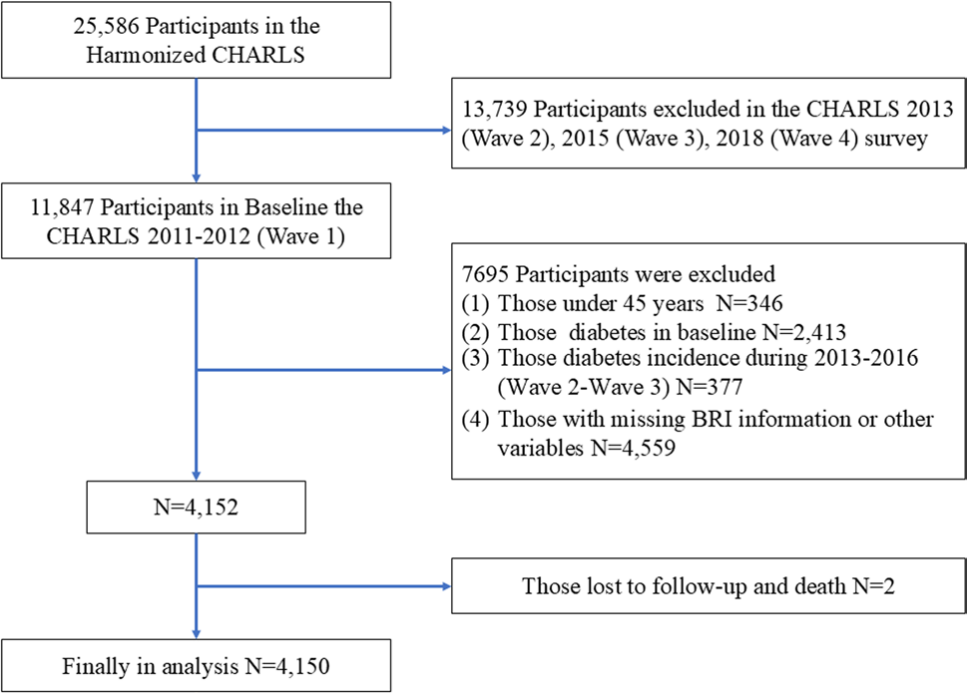
Flow chart of sample selection and the exclusion criteria

In the present study, a total of 25,586 participants were initially sourced from the Harmonized CHARLS database. The exclusion criteria were as follows: first, participants aged ≥ 45 years at the baseline survey (Wave 1) were included. Second, to ensure that all participants were free of DM at the end of the exposure assessment period, we excluded individuals with a prior history of DM during Wave 1 through Wave 3 (2011–2016). Third, participants with insufficient BRI measurements or missing data on other key covariates across Waves 1–3 were excluded. Eligible participants who were alive at the end of Wave 3 constituted the at-risk cohort and were prospectively followed for incident DM events occurring exclusively during Wave 4 (2017–2018). The final analytical sample consisted of 4,150 participants (Fig. 1).

### Blood sample collection and anthropometric assessments

Blood sample collection was carried out under the meticulous supervision of medically trained staff from the Chinese Center for Disease Control and Prevention (CDC). A standardized protocol was followed throughout the process. Specifically, three tubes of venous blood were collected from each participant. Immediately after collection, the samples were transported to local laboratories and stored at 4 °C. These samples were subsequently subjected to centrifugation and then stored at −20 °C. Within two weeks, they were shipped to the central laboratory at the China CDC in Beijing, where they were further preserved at −80 °C until analysis. For the measurement of key blood parameters, such as blood lipids and C-reactive protein (CRP), precise laboratory techniques were employed to ensure accurate results.

Simultaneously, a comprehensive series of anthropometric measurements were conducted. Blood pressure (BP) was measured via an electronic sphygmomanometer (HEM-7200 Monitor; Omron, Kyoto, Japan). The participants were asked to rest in a seated position for 5 min prior to the measurement, and the mean value of three consecutive measurements was used for subsequent analysis. WC was determined by placing a nonstretchable tape measure around the abdomen at the level of the navel during minimal respiration. For height measurement, a 213 stadiometer (Seca GmbH, Hamburg, Germany) was used, with participants standing barefoot on the platform of the instrument. All anthropometric assessments were performed in strict accordance with standardized procedures to guarantee the accuracy and consistency of the collected data [14, 15].

### The definition of the BRI

The BRI is calculated via the following formula [6]:

$$\begin{aligned}&\mathrm{BRI}=364.2-365.5\times\sqrt{1-\frac{\left(WC/2\pi\right)^2}{0.5\times{heigt}^2}},\text{the units of}\\&\text{ measurement for WC and height are centimeters}\left(\mathrm{cm}\right).\end{aligned}$$

### Ascertainment of new-onset DM and onset time

The primary outcome of this study was incident DM, defined as newly self-reported physician-diagnosed cases identified during Wave 4 (2017–2018). Consistent with prior CHARLS studies, diagnosis was based on participants’ affirmative response to the relevant survey question [16, 17]. Participants reporting a new diagnosis at Wave 4, who had no prior self-reported diagnosis in Waves 1 through 3, were classified as incident cases. The time of onset was estimated as the midpoint of the Wave 4 survey period for survival analysis. This ascertainment method was necessitated by the absence of follow-up blood glucose or hemoglobin A1c (HbA1c) measurements in Wave 4. While not equivalent to a biomarker-based diagnosis, self-reported physician-diagnosed DM has been validated as a reliable measure in this and similar cohorts.

### Covariate assessments

In this study, covariate data, including age, sex, education level, marital status, household registration type, smoking and drinking habits, and the history of chronic diseases (such as arthritis, digestive system diseases, heart disease and stroke), were collected at baseline. The education level was classified into three categories: primary school or below, junior high school, and high school or above. Household registration was divided into agricultural and nonagricultural categories. Smoking and drinking statuses were recorded simply as either “yes” or “no”. Hypertension was defined by the following criteria: a systolic blood pressure (SBP) equal to or greater than 140 mmHg, a diastolic blood pressure (DBP) equal to or greater than 90 mmHg, the current use of antihypertensive medication, or a self-reported history of hypertension. BMI was calculated by dividing an individual’s weight in kilograms by the square of their height in meters. The history of chronic diseases was determined on the basis of participants’ self-reported physician diagnoses.

### Statistical analysis

The trajectory is utilized to describe the dynamic process of changes in the factors under investigation over time. In this study, we aimed to group individuals with similar BRI patterns within the CHARLS database from 2011 to 2016. To achieve this goal, we adopted the GBTM to identify the trajectories of the BRI. The trajectory groups were identified based on the probabilistic assignment of each individual’s complete longitudinal data from Waves 1–3, rather than on their baseline value alone. Drawing on the research findings of Nagin [18] and integrating previous studies, we establish a comprehensive approach for determining the optimal model through multiple evaluation criteria. These criteria are detailed as follows: (1) Bayesian information criterion (BIC): This criterion serves to balance the model complexity (in terms of the number of parameters) with its goodness of fit. A lower BIC value indicates a more favorable model, as it implies that the model can explain the data well while maintaining simplicity in its structure. (2) Lo-Mendell-Rubin likelihood ratio test (LMR-LRT): This statistical test is employed to determine the optimal number of groups. A low *p*-value obtained from this test suggests that a model with at least k groups is required to adequately capture the underlying patterns in the data. (3) Entropy: Entropy is used as a measure of classification accuracy. A value closer to 1 indicates a higher level of precision in classifying individuals into different subgroups, making it a commonly used criterion for model selection. (4) Average posterior probability (AvePP): For each subgroup identified by the model, the average posterior probability should be no less than 0.7. This requirement ensures that the model’s assignment of individuals to subgroups is reliable and has a certain level of certainty. (5) Subgroup proportion: The number of individuals within each subgroup should account for at least 5% of the total sample size. This condition helps ensure that each subgroup is adequately represented in the sample and that the model is not overly influenced by small or insignificant subgroups. (6) Model confidence intervals: The confidence intervals for the model should be sufficiently narrow. The narrow confidence intervals indicate a high degree of precision in the model estimates, suggesting that the model can provide relatively accurate predictions within a defined range. (7) BIC difference (△BIC): A larger difference in the BIC between more complex and simpler models favors the acceptance of the more complex model. This criterion helps in comparing different model specifications and selecting the one that strikes the best balance between complexity and explanatory power. (8) Relative entropy (Ek): A value greater than 0.8 for the relative entropy indicates a good model fit, indicating that the model can effectively capture the patterns in the data related to BRI trajectories. After a comprehensive evaluation of the above criteria, we determined that three distinct trajectories were the optimal choice, which start with quadratic shapes and identify. This model was selected because of its statistical significance, excellent fit to the data, and high classification accuracy (Supplementary Tables S1–S3).

With respect to the baseline characteristics of the participants, normally distributed continuous variables are presented as the mean ± standard deviation (*SD*), whereas those with an abnormal distribution are described as the interquartile range (*IQR*). Categorical variables are displayed as frequencies and percentages. To compare the demographic and clinical characteristics among different BRI trajectory groups, for continuous variables, either analysis of variance or the *Kruskal*–*Wallis* test was employed, depending on the specific distribution characteristics. For categorical variables, chi-square tests were utilized. Moreover, we constructed multivariate *Cox* regression models with a pre-specified adjustment strategy to distinguish confounding from potential mediation. Model 1 was adjusted for age and sex. Model 2, our primary confounder-adjusted model, further included socioeconomic and lifestyle factors (education level, marital status, household registration, smoking, and drinking) that are considered common causes of both BRI and DM. Model 3 further incorporated a series of biomarkers—hypertension, triglycerides (TG), high-density lipoprotein cholesterol (HDL-C), low-density lipoprotein cholesterol (LDL-C), uric acid (UA), and CRP—to examine the extent to which the association between BRI trajectories and DM might be mediated through these cardiometabolic pathways.

We constructed receiver operating characteristic (*ROC*) curves to evaluate the discriminatory power of the baseline BRI, BMI, and WC for incident DM. The areas under the *ROC* curves were compared via DeLong’s test.

To explore the additional contributions that the BRI makes to DM risk assessment, over and above those of traditional risk factors, we carry out a further evaluation of the capacity of BRI trajectories to reclassify risk within models that incorporate traditional risk factors. Specifically, this assessment was performed by employing two methods: net reclassification improvement (NRI) and integrated discrimination improvement (IDI) [19].

Within the framework of the multivariate *Cox* proportional hazards regression model, we performed subgroup analyses on the basis of both demographic characteristics and health-related indicators. The aim of these analyses was to further investigate the associations between BRI trajectories and the risk of DM. To verify the robustness of our model, a series of sensitivity analyses were conducted. First, we examined the potential impact of participants’ use of BP and lipid-lowering medications. Second, and as pre-specified in our statistical plan, we performed a comprehensive adjustment for a history of various chronic diseases in a separate model to test whether the observed associations were independent of these conditions. The statistical data analysis was conducted via *Stata/MP* 18.0 and *R* version 4.4.1 software. A *P*-value of less than 0.05 was regarded as statistically significant (two-tailed).

## Results

### Baseline characteristics

**Fig. 2:**
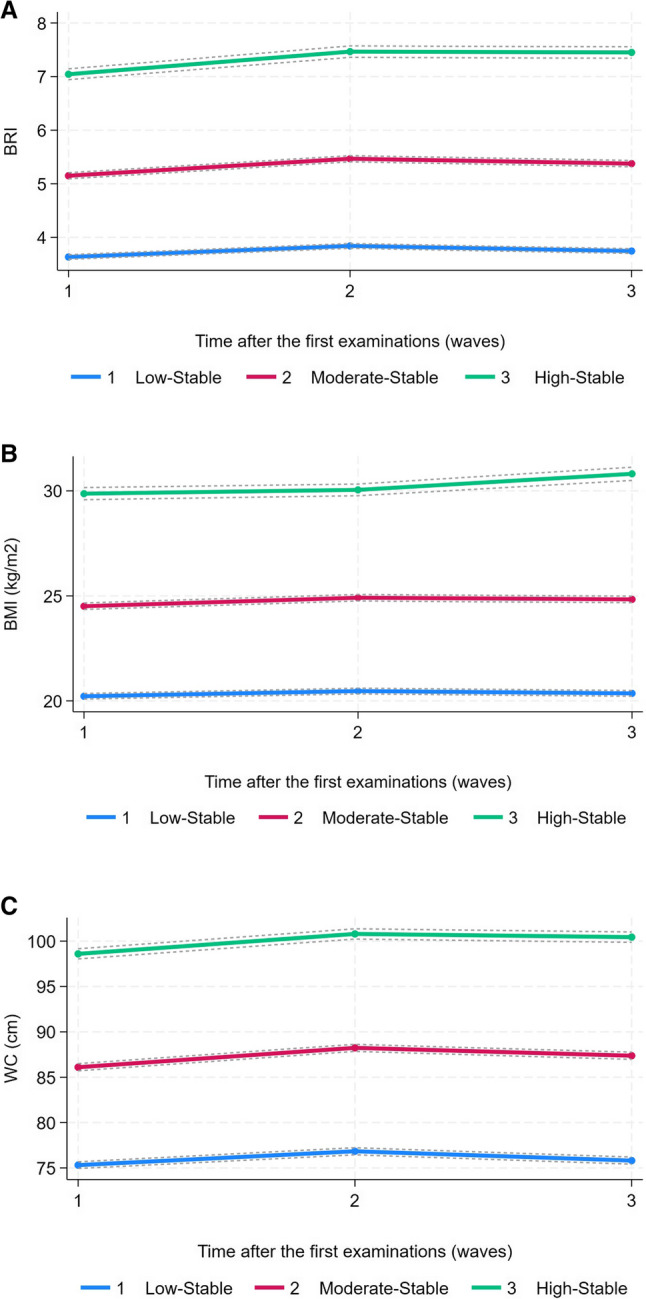
**A** GBTM - derived BRI trajectory models in the three groups in CHARLS. Solid lines represent the estimated mean trajectory for each group; dashed lines represent the 95% confidence intervals around each mean trajectory. **B** GBTM - derived BMI trajectory models in the three groups in CHARLS. Solid lines represent the estimated mean trajectory for each group; dashed lines represent the 95% confidence intervals around each mean trajectory. **C** GBTM - derived WC trajectory models in the three groups in CHARLS. Solid lines represent the estimated mean trajectory for each group; dashed lines represent the 95% confidence intervals around each mean trajectory

A total of 4,150 participants were enrolled in this study, among whom 1,935 were males and 2,215 were females. The detailed baseline characteristics of the participants are presented in Table 1. The application of GBTM to the longitudinal BRI data from Waves 1 to 3 (2011–2016) identified three distinct trajectory patterns, which optimally captured the heterogeneity in the population’s body roundness evolution over this period. These model-derived patterns were characterized by different levels of statistical stability and were thus designated as the low-stable (*n* = 2,032, 48.96%), moderate-stable (*n* = 1,714, 41.30%), and high-stable (*n* = 404, 9.73%) groups (Fig. 2 A). This classification indicates that an individual’s risk profile is defined by their sustained exposure level to a specific body roundness phenotype throughout the 6-year modeling period. When the high-stability group was compared with the low-stability group, several differences were observed. The high stability-group had a relatively lower proportion of males, a lower level of educational attainment, and lower rates of smoking and drinking. Nevertheless, this group presented a greater incidence of arthritis, dyslipidemia, heart disease, and stroke. With respect to physiological parameters, the high stability group had higher levels of SBP and DBP, total cholesterol (CHO), TG, LDL-C, CRP, and BMI.

**Table 1:** Baseline characteristics of participants stratified by BRI trajectory groups

Variable	Low-stable (*N* = 2,032)	Moderate-stable (*N* = 1,714)	High-stable (*N* = 404)	*P* value
Demographic factors				
Age, years	58.66 ± 8.77	58.56 ± 8.74	59.48 ± 8.87	0.160
Sex, *n* (%)				
Male	1,253 (61.7%)	623 (36.3%)	59 (14.6%)	< 0.001
Female	779 (38.3%)	1,091 (63.7%)	345 (85.4%)	
Education level, *n* (%)				
Primary school or below	1,419 (69.8%)	1,232 (71.9%)	317 (78.5%)	0.006
Middle school	404 (19.9%)	323 (18.8%)	65 (16.1%)	
High school or above	209 (10.3%)	159 (9.3%)	22 (5.4%)	
Marital status				
No	2,017 (99.3%)	1,709 (99.7%)	404 (100.0%)	0.053
Yes	15 (0.7%)	5 (0.3%)	0 (0.0%)	
Household registration				
Agricultural	1,786 (87.9%)	1,428 (83.3%)	352 (87.1%)	< 0.001
Non-agricultural	246 (12.1%)	286 (16.7%)	52 (12.9%)	
Lifestyle factors				
Drinking, *n* (%)				
None	1,100 (54.1%)	1,155 (67.4%)	320 (79.2%)	< 0.001
Yes	932 (45.9%)	559 (32.6%)	84 (20.8%)	
Smoking, *n* (%)				
No	976 (48.0%)	1,202 (70.1%)	336 (83.2%)	< 0.001
Yes	1,056 (52.0%)	512 (29.9%)	68 (16.8%)	
Comorbidities				
Arthritis, *n* (%)	622 (30.6%)	593 (34.6%)	169 (41.8%)	< 0.001
Dyslipidemia, *n* (%)	85 (4.2%)	150 (8.9%)	53 (13.5%)	< 0.001
Digestive disease, *n* (%)	501 (24.7%)	373 (21.8%)	71 (17.6%)	0.003
Heart disease, *n* (%)	189 (9.3%)	212 (12.4%)	49 (12.1%)	0.007
Stroke, *n* (%)	34 (1.7%)	34 (2.0%)	15 (3.7%)	0.037
Clinical features				
Systolic, mmHg	125.21 ± 19.86	131.41 ± 21.13	137.29 ± 21.25	< 0.001
Diastolic, mmHg	72.85 ± 11.76	76.53 ± 12.04	80.17 ± 12.38	< 0.001
TG, mg/dL	88.50 (64.61–124.79)	111.51 (80.54–161.96)	127.00 (94.69–170.80)	< 0.001
CHO, mg/dL	187.19 ± 35.92	196.23 ± 37.03	201.87 ± 38.39	< 0.001
HDL-C, mg/dL	55.68 ± 15.57	49.60 ± 13.86	47.11 ± 12.50	< 0.001
LDL-C, mg/dL	112.41 ± 31.26	120.94 ± 34.46	124.00 ± 36.71	< 0.001
UA, mg/dL	4.39 ± 1.20	4.41 ± 1.23	4.44 ± 1.16	0.740
CRP, mg/L	0.76 (0.44–1.56)	1.05 (0.61–2.09)	1.54 (0.86–3.01)	< 0.001
BMI, kg/m^2^	20.92 ± 2.65	24.66 ± 2.72	28.62 ± 3.55	< 0.001

*Abbreviations*: *TG* triglycerides, *CHO* total cholesterol, *HDL-C* high-density lipoprotein cholesterol, *LDL-C* low-density lipoprotein cholesterol, *UA* uric acid, *CRP* C-reactive protein, *BMI* body mass index

### Associations between BRI trajectories and DM incidence

During the 2017–2018 (wave 4) follow-up, a total of 103 incident DM cases were identified. Specifically, 28 cases (1.38%) were identified in the low-stability group, 52 cases (3.03%) in the moderate stability group, and 23 cases (5.69%) in the high stability group. As detailed in Table 2, the multivariate *Cox* regression analysis revealed a strong, graded association between BRI trajectories and the risk of incident diabetes. In our primary confounder-adjusted model (Model 2), participants in the high-stable group had a significantly elevated risk of diabetes compared to those in the low-stable group (hazard ratio [*HR*] = 4.33, 95% confidence interval [*CI*]: 2.41–7.77). A significant but attenuated association was still observed in the mediation exploration model (Model 3), which further adjusted for cardiometabolic biomarkers (*HR* = 2.63, 95% *CI*: 1.41–4.91).

**Table 2:** Association between BRI trajectories and incident DM

BRI Trajectory Group	Incident DM cases, *n* (%)	Model 1^a^	Model 2^b^	Model 3^c^
Low-stable	28 (1.38)	1.00 (Reference)	1.00 (Reference)	1.00 (Reference)
Moderate-stable	52 (3.03)	2.21 (1.38–3.55)	2.20 (1.37–3.54)	1.64 (1.01–2.68)
High-stable	23 (5.69)	4.28 (2.38–7.68)	4.33 (2.41–7.77)	2.63 (1.41–4.91)
*P* for trend		< 0.001	< 0.001	< 0.010

^a^Model 1 adjusted for age, sex

^b^Model 2 further adjusted for further adjusting for education level, marital status, household status, drinking status and smoking status based on model 1

^c^Model 3 further adjusted for hypertension, TG, HDL-C, LDL-C, UA and CRP based on model 2

### Comparison of predictive efficacy between BRI and traditional obesity indices (BMI/WC)

**Fig. 3:**
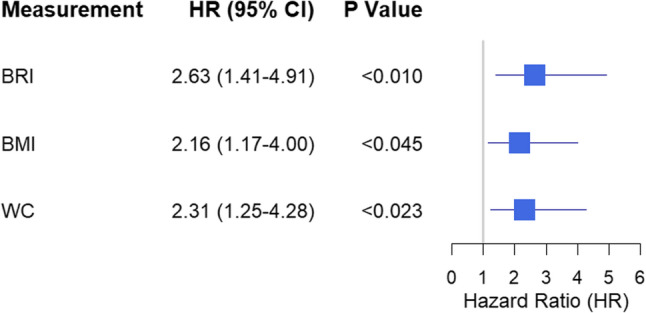
*HR* and 95% CI for new-onset diabetes in the high-stable group across different adiposity indices

To evaluate the predictive value of the BRI relative to traditional obesity indices, we conducted a comparative analysis from two perspectives. First, at the single-time-point measurement level, we compared the discriminatory ability of the baseline BRI, BMI, and WC for DM incidence using *ROC* curve analysis. As shown in Supplementary Fig. 1, the *ROC* curve analysis revealed that the discriminatory power of a single baseline BRI measurement for incident DM was comparable to that of BMI and WC (AUC for BRI: 0.63 vs. BMI: 0.64 vs. WC: 0.63; Delong’s test, all *P* > 0.05), highlighting the limitation of cross-sectional assessment. Second, at the longitudinal trajectory level, we applied the same GBTM methodology used for BRI to construct parallel trajectory models for BMI and WC (Fig. 2 B and C). *Cox* regression analysis based on GBTM revealed that in the high-stability group, BRI had the highest hazard ratio: BRI: *HR* = 2.63 (95% *CI*: 1.41–7.77); BMI: *HR* = 2.16 (95% *CI*: 1.17–4.00); WC: *HR* = 2.31 (95% *CI*: 1.25–4.28) (Table 3; Fig. 3). It is important to note that the confidence intervals for the high-stable groups of BRI, BMI, and WC showed substantial overlap, indicating that the risk estimates for these indices were not statistically distinguishable from one another. Finally, to quantify the additive predictive value of BRI trajectories, we calculated the NRI and IDI. The results demonstrated that adding BRI trajectories to the conventional risk model yielded a statistically significant, though modest, improvement in both reclassification (NRI = 10.76%, 95% *CI*: 2.40–19.47) and discrimination (IDI = 0.27%, 95% *CI*: 0.03–0.52). To systematically evaluate and compare the additive predictive value across indices, parallel NRI and IDI analyses were conducted for BMI and WC trajectories. Notably, the addition of the BMI trajectory did not result in a significant improvement in either NRI (3.73%, 95% *CI*: −4.11–11.35; *P* = 0.340) or IDI (0.17%, 95% *CI*: 0.03–0.36; *P* = 0.090). Although the WC trajectory also showed significant improvement, its point estimates for both NRI and IDI were lower than those of BRI. These comparative results are detailed in Table 4.

**Table 3:** *HR* for incident DM according to BMI and WC trajectory groups

Index & Trajectory Group	Incident DM cases, *n* (%)	Model 1^a^	Model 2^b^	Model 3^c^
BMI trajectories				
Low-stable	26 (1.45)	1.00 (Reference)	1.00 (Reference)	1.00 (Reference)
Moderate-stable	53 (2.73)	1.81 (1.12–2.91)	1.82 (1.12–2.93)	1.35 (0.82–2.22)
High-stable	24 (5.67)	3.65 (2.07–6.46)	3.70 (2.08–6.56)	2.16 (1.17–4.00)
*P* for trend		< 0.001	< 0.001	0.045
WC trajectories				
Low-stable	19 (1.29)	1.00 (Reference)	1.00 (Reference)	1.00 (Reference)
Moderate-stable	51 (2.53)	1.91 (1.13–3.24)	1.93 (1.14–3.92)	1.50 (0.87–2.58)
High-stable	33 (4.99)	3.80 (2.16–6.68)	3.86 (2.18–6.82)	2.31 (1.25–4.28)
* P* for trend		< 0.001	< 0.001	0.023

^a^Model 1 adjusted for age, sex

^b^Model 2 further adjusted for further adjusting for education level, marital status, household status, drinking status and smoking status based on model 1

^c^Model 3 further adjusted for hypertension, TG, HDL-C, LDL-C, UA and CRP based on model 2

**Table 4:** Incremental predictive value of adding adiposity index trajectories to a conventional risk model for DM

	Continuous NRI (95% CI), %	*P* value	IDI (95%CI), %	*P* value
BRI				
Conventional model	Reference		Reference	
Conventional model + trajectory of BRI	10.76 (2.40–19.47)	0.014	0.27 (0.03–0.52)	0.039
BMI				
Conventional model	Reference		Reference	
Conventional model + trajectory of BMI	3.73 (−4.11–11.35)	0.340	0.17 (0.03–0.36)	0.090
WC				
Conventional model	Reference		Reference	
Conventional model + trajectory of WC	9.48 (1.85–17.66)	0.028	0.24 (0.05–0.44)	0.018

Conventional model included age, sex, education level, marital status, household status, drinking status, smoking status, hypertension, TG, HDL-C, LDL-C, UA and CRP

*BRI* body roundness index, *BMI* body mass index, *WC* waist circumference, *DM* diabetes mellitus, *NRI* net reclassification improvement; IDI, integrated discrimination index

### Subgroup and sensitivity analysis

**Fig. 4:**
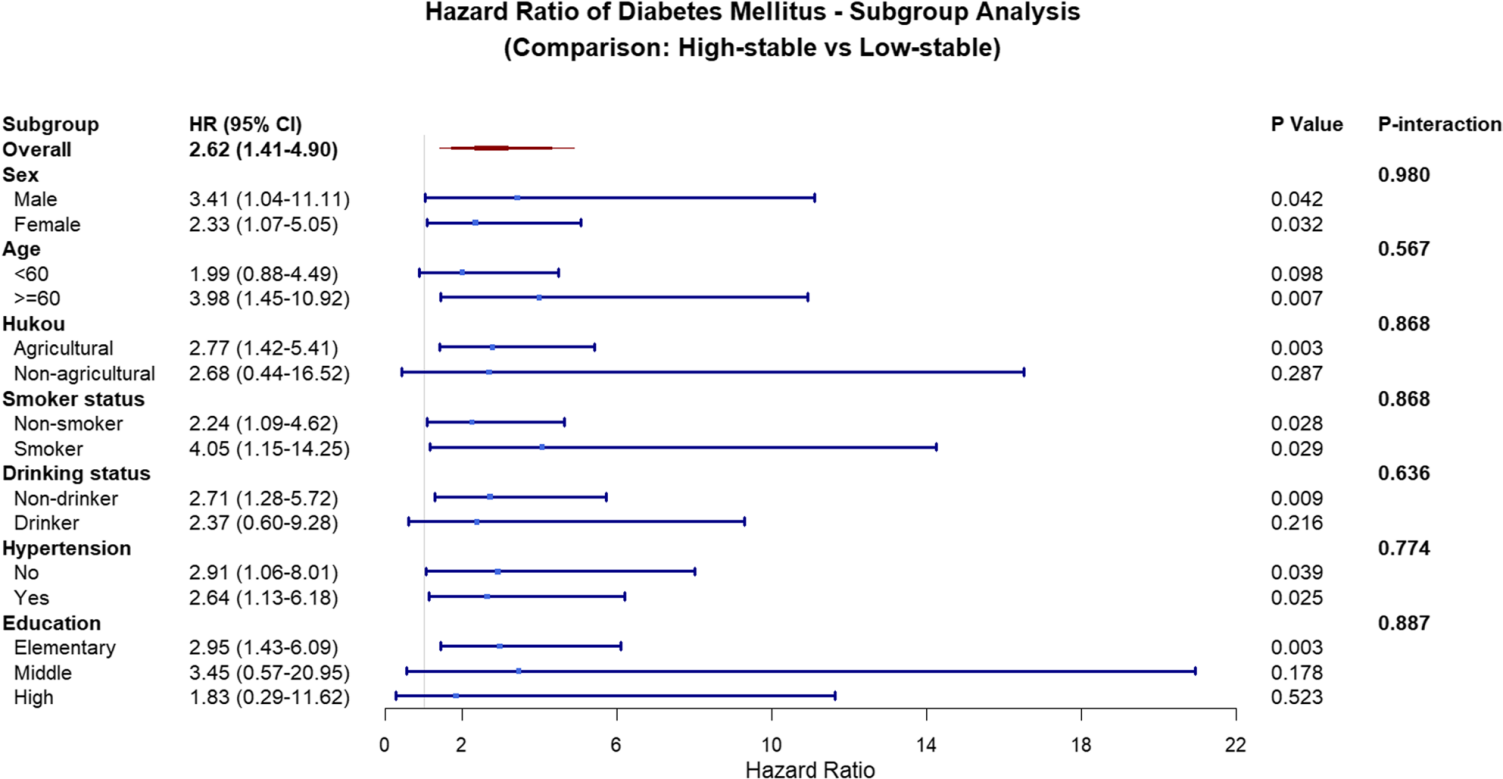
Subgroup analysis of the association between BRI trajectories and incident diabetes mellitus

In the subgroup analysis, the association between the high-stable trajectory group (compared with the low-stable group) and an increased risk of DM showed a consistent trend across most population characteristics. The point estimates of the hazard ratios were relatively higher in individuals aged ≥ 60 years (*HR* = 3.98, 95% *CI*: 1.45–10.92), males (*HR* = 3.41, 95% *CI*: 1.04–11.11), and current smokers (*HR* = 4.05, 95% *CI*: 1.15–14.25). Although the risk estimates did not reach statistical significance in some subgroups (e.g., non-agricultural household registration, drinkers, and those with middle or higher education levels), all interaction tests were non-significant (all *P*-interaction > 0.05). This indicates that the association between the two trajectory groups did not significantly differ across populations with different sex, age, behavioral, or socioeconomic characteristics, supporting the robustness of the main findings in diverse subgroups (Fig. 4). Our team further conducted a series of sensitivity analyses to verify the robustness of the results. First, we examined the potential impact of concomitant medications by including and excluding patient groups receiving treatment for hypertension and dyslipidemia, which confirmed the consistency of the findings (Supplementary Tables S4–S5). Furthermore, we performed a comprehensive adjustment for a history of various chronic diseases (Model 4), which yielded results highly consistent with our primary analysis (High-stable vs. Low-stable: *HR* = 2.78, 95% *CI*: 1.48–5.22; see Supplementary Table S6 for full details). Collectively, these sensitivity analyses underscore the robustness of the observed association between BRI trajectories and DM incidence.

## Discussion

This study utilized longitudinal data from the CHARLS and GBTM to explore the association between long-term trajectories of the BRI and the risk of new-onset DM. The results demonstrate that maintaining a persistently high BRI trajectory is an independent risk factor for DM incidence, and that longitudinal trajectory analysis provides methodological value for risk stratification beyond traditional single-time-point measurements.

### Key findings and interpretation of trajectory stability

Analysis of data from 2011 to 2016 revealed three primary, stable trajectory patterns of BRI evolution among middle-aged and older Chinese adults: low-stable, moderate-stable, and high-stable. This finding suggests that an individual’s long-term DM risk phenotype is largely determined by the BRI level sustained during this period. It is important to note that the core patterns identified by trajectory modeling were characterized as “stable,” indicating that the primary driver of DM risk in this cohort was sustained exposure to a particular level of body roundness, rather than specific dynamic change [11–13, 18]. However, this precisely highlights the clinical utility of the present study: GBTM offers a data-driven, longitudinally validated method for risk stratification. This approach confirmed that individuals probabilistically assigned to the “high-stable” group based on multi-wave data represent a validated, persistently high-risk phenotype. Compared to a single baseline assessment, this method provides a more robust means for targeted prevention [18, 20]. In other words, the very ‘stable’ nature of the trajectories indicates that, in this cohort, an individual’s DM risk phenotype is primarily defined by their long-term maintained level of body morphology, rather than being driven by specific dynamic change patterns.

### From static association to dynamic risk identification: methodological advancement

Although cross-sectional studies have established an association between BRI and DM risk [7, 21], they cannot reveal the dynamic evolution of risk factors. By applying longitudinal trajectory modeling, this study shifts the analytical perspective from “static association” to “dynamic risk identification.” Its core contribution lies in applying GBTM within a nationally representative middle-aged and older adult cohort (CHARLS) to demonstrate that a long-term “stable trajectory” of high BRI constitutes a stronger signal of DM risk than a single high BRI measurement [7, 21, 22]. This is not a mere replication of cross-sectional associations but provides a more robust risk assessment paradigm that incorporates a temporal dimension.

### Comparison and synergistic value of BRI versus traditional obesity indices

At the single-time-point measurement level, *ROC* curve analysis showed comparable discriminatory power of baseline BRI, BMI, and WC for predicting future DM (AUC: 0.63–0.64). However, analysis at the longitudinal trajectory level offered deeper insights. Although the point estimate for the *HR* was numerically highest for the BRI high-stable group, its confidence interval substantially overlapped with those of the BMI and WC high-stable groups (Table 3). This indicates that while all three indices robustly identify a high-risk phenotype, their risk estimates are not statistically distinguishable in this cohort. Therefore, a more significant finding of this study may lie at the methodological level: irrespective of whether BRI, BMI, or WC is used, longitudinal trajectory modeling itself, by integrating multi-time-point information, provides more robust risk identification than a single measurement. NRI analysis quantified this advantage: adding BRI trajectories to a conventional risk model resulted in significant reclassification improvement (NRI = 10.76%), with its point estimate exceeding that for WC trajectories, while the improvement for BMI trajectories was not statistically significant. These results underscore that the primary contribution of this study is the validation of the longitudinal trajectory paradigm as a superior tool for risk stratification. Its clinical utility lies in more accurately reclassifying individuals, particularly those at intermediate risk, thereby enabling more efficient allocation of preventive resources beyond the limitations of a single cross-sectional measurement [19].

### Association strength, mechanism exploration, and adjustment strategy

A strong association between BRI trajectories and DM was evident after adjusting for demographic factors like age and sex (Model 1). After further adjustment for socioeconomic and lifestyle confounders such as education, marital status, household registration, smoking, and alcohol consumption (Model 2, representing the primary confounder-adjusted model), the *HR* for the high-stable versus low-stable group was 4.33. Subsequent incorporation of biomarkers including hypertension, blood lipids, UA, and CRP in Model 3 attenuated the *HR* to 2.63. This attenuation from Model 2 to Model 3 suggests that BRI may partially mediate DM risk through cardiometabolic pathways involving blood lipids and BP [23–29]. As an indicator reflecting visceral adiposity and body shape, the association between BRI and DM may be linked to mechanisms related to abdominal obesity, such as insulin resistance, a chronic inflammatory state, and associated metabolic disorders [6, 23–29].

### Study strengths and limitations

This study benefits from a nationally representative, large-sample longitudinal cohort, standardized measurements, and adjustment for a wide range of covariates, lending good internal validity to the conclusions [14, 16]. Several limitations must also be acknowledged. First, the identified trajectories were primarily stable; their number and shape may be influenced by measurement time points and modeling parameters, and the sample size of the high-stable group was relatively small [30]. Second, DM diagnosis was based on self-report, which, although validated in similar cohorts [31, 32], may be subject to misreporting. Third, potential confounders such as physical activity were not adjusted for. Fourth, the study population was limited to middle-aged and older Chinese adults, necessitating caution in generalizing the findings. Finally, the observational design precludes causal inference.

### Practical implications

The findings of this study offer clear practical implications for DM prevention. In clinical and public health settings, utilizing routinely available height and WC data from physical examinations to calculate the BRI and observing its trajectory over several years enables low-cost, efficient identification of individuals exhibiting “long-term high body roundness risk.” Compared to intervention thresholds based on single measurements, this dynamic identification strategy allows for more precise targeting of individuals who would benefit most from intensive lifestyle interventions or close monitoring, thereby optimizing the allocation of healthcare resources and facilitating earlier intervention [7, 21, 33].

In summary, this study not only confirms that maintaining a high BRI trajectory is a significant risk factor for DM but, more importantly, validates longitudinal trajectory modeling as a powerful tool. This tool can transcend single-time-point measurements to more reliably identify individuals with sustained high risk, thereby providing a novel perspective and an evidence-based foundation for early and targeted DM prevention in the population.

## Conclusion

This study demonstrates a significant association between the BRI trajectories and the risk of DM incidence, confirming that a sustained high-BRI trajectory is an important risk factor. More importantly, by employing trajectory modeling, this research validates the value of a longitudinal analytical framework for risk identification. This approach, which integrates multi-timepoint information, provides a more robust basis for the early identification of high-risk individuals compared to a single measurement. Future studies should further validate these findings in broader populations and explore the underlying mechanisms to support more targeted diabetes prevention strategies.

## Supplementary Information

Supplementary Material 1

## Data Availability

The datasets analyzed in this study are publicly archived in the CHARLS repository (http://charls.pku.edu.cn/en/). Access requires registration as an authorized user in accordance with the CHARLS data access protocol.
