# Supplementary material for: Association between body roundness index trajectories and the incidence of diabetes mellitus: a perspective from the China health and retirement longitudinal study
Source: Lipids Health Dis. 2025 Dec 30;25:32. doi: 10.1186/s12944-025-02840-y (PMC12860073; doi:10.1186/s12944-025-02840-y)

**Table S1 Indices for Assessing the Fit of Various Trajectory Models**

|  |  |  | The posterior probability of  group members, Pj (%) | Actual group membership  probability,πj(%) |  |  |  |
| --- | --- | --- | --- | --- | --- | --- | --- |
| Group (Parameter) | AvePP (%) | OCC |  |  | BIC | △BIC | Ej |
| 2 Group (2 2) | 96.03-92.13 | 68.46-31.54 | 65.99-28.82 | 68.72-31.28 | -19428.48 |  | 0.824 |
| 3 Group (2 2 2) | 93.38-91.93-92.43 | 48.23-41.95-9.83 | 45.72-37.97-9.00 | 48.96-41.30-9.73 | -18276.64 | 1151.84 | 0.841 |
| 4 Group (2 2 2 2) | 91.30-87.30-8.36-0 | 35.45-41.46-19.35-3.74 | 32.28-36.82-1.58-0 | 35.35-42.17-18.82-3.66 | -17805.05 | 471.59 | 0.821 |

**Table S2** **Estimation of the parameters for the optimal group trajectory model**

|  |  |  |  | The posterior probability of  group members, Pj (%) | Actual group membership  probability,πj(%) |
| --- | --- | --- | --- | --- | --- |
| Group | Class | AvePP (%) | OCC |  |  |
| 3 Group (2 2 2) | Class1 | 93.38 | 48.23 | 45.72 | 48.96 |
|  | Class2 | 91.93 | 41.95 | 37.97 | 41.30 |
|  | Class3 | 92.43 | 9.83 | 9.00 | 9.73 |

**Table S3 Evaluating the fit of each trajectory group within the trajectory model**

| Group | Class | Parameter | β | SE | t | P |
| --- | --- | --- | --- | --- | --- | --- |
| 3 Group (2 2 2) | Class1 | Intercept | 3.63462 | 0.02172 | 167.362 | < 0.0001 |
|  |  | Linear | 0.35766 | 0.04782 | 7.479 | < 0.0001 |
|  |  | Quadratic | -0.15105 | 0.02298 | -6.573 | < 0.0001 |
|  | Class2 | Intercept | 5.15025 | 0.02909 | 177.048 | < 0.0001 |
|  |  | Linear | 0.52201 | 0.05205 | 10.030 | < 0.0001 |
|  |  | Quadratic | -0.20446 | 0.02504 | -8.166 | < 0.0001 |
|  | Class3 | Intercept | 7.04327 | 0.05172 | 136.172 | < 0.0001 |
|  |  | Linear | 0.64020 | 0.10689 | 5.989 | < 0.0001 |
|  |  | Quadratic | -0.21832 | 0.05141 | -4.247 | < 0.0001 |

**Table S4** **Sensitivity analysis considering medications history for association of BRI trajectories with new-onset DM in CHARLS (2011-2018)**

| BRI Trajectory Group | Incident DM cases, n (%) | Model 1^a^ | Model 2^b^ | Model 3^c^ | Model 4^d^ |
| --- | --- | --- | --- | --- | --- |
| Low-stable  Moderate-stable  High-stable  *P* for trend | 28 (1.38)  52 (3.03)  23 (5.69) | 1.00 (Reference)  2.21 (1.38-3.55)  4.28 (2.38-7.68)  < 0.001 | 1.00 (Reference)  2.20 (1.37-3.54)  4.33 (2.41-7.77)  < 0.001 | 1.00 (Reference)  1.64 (1.01-2.68)  2.63 (1.41-4.91)  < 0.010 | 1.00 (Reference)  1.62 (1.00-2.66)  2.65 (1.42-4.95)  < 0.010 |

^a^Model 1 adjusted for age, sex.

^b^Model 2 further adjusted for further adjusting for education level, marital status, household status, drinking status and smoking status based on model 1.

^c^Model 3 further adjusted for hypertension, TG, HDL-C, LDL-C, UA and CRP based on model 2.

^d^Model 4 further adjusted for taking medications for hypertension and dyslipidemia based on model 3.

**Table S5 Sensitivity analysis excluded patients with treated hypertension and dyslipidemia (N=752) to assess the association of BRI trajectories with new-onset DM in the CAHRLS cohort (2011-2018)**

| BRI Trajectory Group | Incident DM cases, n (%) | Model 1^a^ | Model 2^b^ | Model 3^c^ |
| --- | --- | --- | --- | --- |
| Low-stable  Moderate-stable  High-stable  *P* for trend | 25 (1.38)  31 (2.36)  14 (5.15) | 1.00 (Reference)  1.69 (0.98-2.91)  2.73 (1.86-7.47)  0.001 | 1.00 (Reference)  1.70 (0.98-2.93)  3.81 (1.90-7.63)  < 0.001 | 1.00 (Reference)  1.33 (0.76-2.34)  2.54 (1.23-5.26)  0.040 |

^*^is sensitivity analysis with excluded treated hypertension and dyslipidemia patients (N=752)

^a^Model 1 adjusted for age, sex.

^b^Model 2 further adjusted for further adjusting for education level, marital status, household status, drinking status and smoking status based on model 1.

^c^Model 3 further adjusted for hypertension, TG, HDL-C, LDL-C, UA and CRP based on model 2.

**Table S6** **Sensitivity analysis adjusting for chronic comorbidities for association of BRI trajectories with new-onset DM in CHARLS (2011-2018)**

| BRI Trajectory Group | Incident DM cases, n (%) | Model 1^a^ | Model 2^b^ | Model 3^c^ | Model 4^d^ |
| --- | --- | --- | --- | --- | --- |
| Low-stable  Moderate-stable  High-stable  *P* for trend | 28 (1.38)  52 (3.03)  23 (5.69) | 1.00 (Reference)  2.21 (1.38-3.55)  4.28 (2.38-7.68)  < 0.001 | 1.00 (Reference)  2.20 (1.37-3.54)  4.33 (2.41-7.77)  < 0.001 | 1.00 (Reference)  1.64 (1.01-2.68)  2.63 (1.41-4.91)  < 0.010 | 1.00 (Reference)  1.64 (1.00-2.69)  2.78 (1.48-5.22)  < 0.010 |

^a^Model 1 adjusted for age, sex.

^b^Model 2 further adjusted for further adjusting for education level, marital status, household status, drinking status and smoking status based on model 1.

^c^Model 3 further adjusted for hypertension, TG, HDL-C, LDL-C, UA and CRP based on model 2.

^d^Model 4 further adjusted for a history of various chronic diseases based on model 3.

**Supplementary Figure 1***ROC* curves and area under the AUC comparison for BRI, BMI, and WC in predicting incident DM


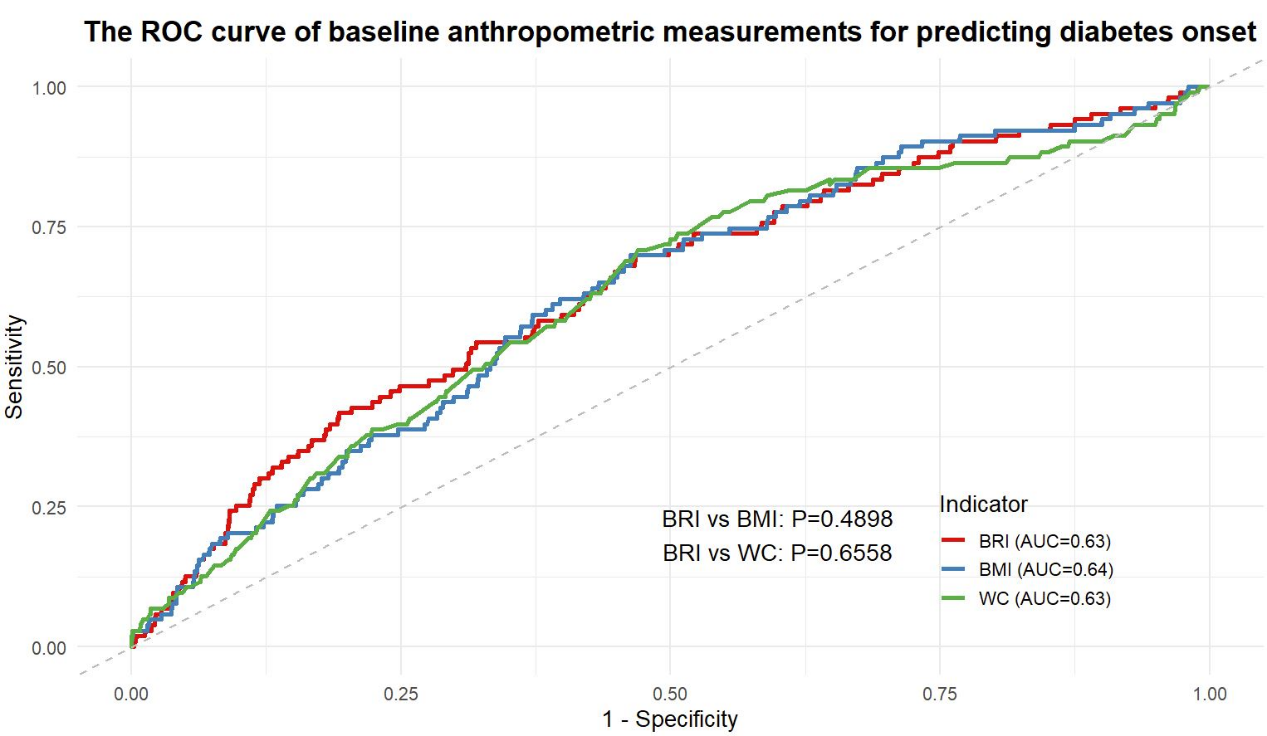

Supplement: Supplementary file 1 — Supplementary Material 1 [file 12944_2025_2840_MOESM1_ESM.docx]
